# A SERS-Based Dual-Parameter Monitoring Nanoprobe of ROS and PI3K/Akt during Ginsenoside Rg3-Induced Cell Apoptosis

**DOI:** 10.3390/bios13020212

**Published:** 2023-01-31

**Authors:** Jianhui Wan, Wendai Cheng, Xinyue Xing, Yuting He, Ping Tang, Yaping Feng, Shengde Liu, Xiaoxu Lu, Liyun Zhong

**Affiliations:** 1 Guangdong Provincial Key Laboratory of Nanophotonic Functional Materials and Devices, South China Normal University, Guangzhou 510006, China; 2 Guangdong Provincial Key Laboratory of Photonics Information Technology, Guangdong University of Technology, Guangzhou 510006, China

**Keywords:** surface-enhanced Raman scattering (SERS), dual-parameter monitoring probe, reactive oxygen species (ROS), PI3K/Akt pathway, cell apoptosis, ginsenosides Rg3

## Abstract

Both the reactive oxygen species (ROS) level and Phosphatidylinositol 3 Kinase (PI3K) protein content are two crucial parameters for characterizing states of cell apoptosis. Current methods measure these parameters with two different techniques, respectively, which usually lead to evaluation contingency. Ginsenoside Rg3 exhibits an excellent anticancer effect, which is enacted by the Phosphatidylinositol 3 Kinase/Protein Kinase B (PI3K/Akt) pathway involving ROS; however, the precise mechanism that induces cell apoptosis remains unknown. This is due to the lack of information on quantitative intracellular ROS and PI3K. Here, we used a surface-enhanced Raman scattering (SERS)-based boric acid nanoprobe to monitor the intracellular ROS level and phosphatidylinositol-3,4,5-triphosphate (PI(3,4,5)P_3_) content, which reflects the regulatory effect of the PI3K/Akt pathway. After treatment with ginsenoside Rg3, the PI3K/Akt content first increased and then decreased as the ROS level increased. Moreover, when the ROS level significantly increased, the mitochondrial membrane potential reduced, thus indicating the dynamic regulation effect of intracellular ROS level on the PI3K/Akt pathway. Importantly, in addition to avoiding evaluation contingency, which is caused by measuring the aforementioned parameters with two different techniques, this SERS-based dual-parameter monitoring nanoprobe provides an effective solution for simultaneous ROS level and PI3K content measurements during cell apoptosis. Furthermore, the intracellular ROS level was also able to have a dynamic regulatory effect on the PI3K/Akt pathway, which is essential for studying ROS/PI3K/Akt-pathway-related cell apoptosis and its activation mechanism.

## 1. Introduction

Reactive oxygen species (ROS) are byproducts of cellular metabolism produced in the cytoplasm, mitochondria, endoplasmic reticulum, and other organelles. ROS have been developed as a valuable parameter for characterizing cell apoptosis state [1,2,3,4,5]. ROS-related cell apoptosis or activation is implemented with a signaling pathway, and the proper balance between self-renewal and differentiation is essential for cell survival [6,7]. Additionally, the ROS regulatory effect is closely related to the ROS level and cell type [8], and the PI3K/Akt pathway that uses ROS plays a critical regulatory role in proliferation, growth, survival, and migration [9,10]. In addition to regulating cell function, the PI3K/Akt pathway also reveals an inhibitory effect of tumor occurrence [11,12], autophagy regulation, and response to ROS level [13]. PI3K/Akt pathway-based inhibition is also an effective solution that is used in cancer therapies involving ROS regulation [14].

Ginsenoside Rg3, one of the most active ingredients in ginseng, exhibits an evident anticancer effect [15,16,17]. By acting on the G2 phase of the cell division cycle, Rg3 can inhibit the synthesis of early mitotic proteins and ATP, which then slows down cellular proliferation and growth [18]. The regulatory effect of ginsenoside on the ROS/PI3K/Akt pathway has been reported [19,20]. By restoring the upstream PI3K/Akt pathway using d-galactose, Rg3 is able to attenuate the apoptosis of liver cells and kidney cells [21]. Through ROS/PI3K/Akt pathway inhibition, ginsenoside is also able to reduce the viability of MCF-7 cells [19]. Due to the abnormal performance in tumors, the PI3K/Akt pathway is becoming an attractive target for developing anticancer therapies; however, its specific regulatory mechanism remains unknown.

Recent studies have also shown that Rg3-induced ROS levels change in accordance with cell type [17]. After Rg3 treatment, the ROS production in MCF-7 cells and Lewis lung carcinoma (LLC) cells has an inhibitory [19,22], but promotional effect in Hela cells through downregulation of PI3K/Akt [16]. Consequently, the intracellular ROS level is a crucial parameter for studying the mechanism of Rg3-induced cell apoptosis.

**Scheme 1:**
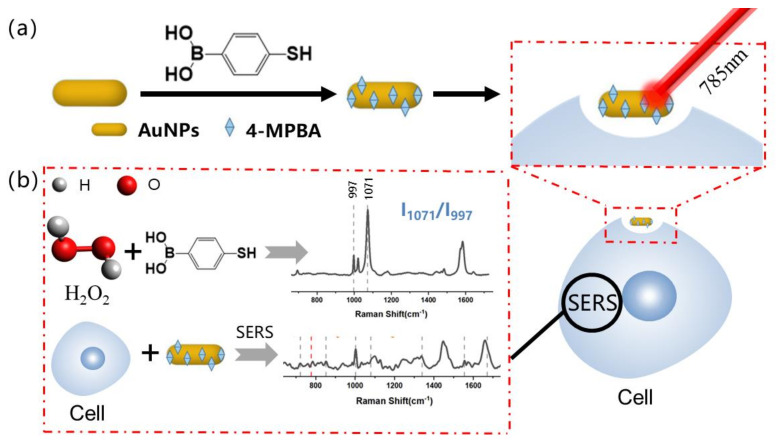
(**a**) Preparation of boronic acid based H_2_O_2_ nanoprobe; (**b**) synergistic effect of SERS detection of PI3K and H_2_O_2_.

In existing methods, intracellular ROS level and PI3K/Akt content are measured using a fluorescent probe 2,7-Dichlorodihydrofluorescein Diacetate (DCFH-DA) [10] and Western blot analysis [12,13,23,24], respectively. Although flow cytometry (FCM) may also be used [25], preparing samples using this method can be troublesome. Recently, gold or silver nanoprobes have been developed as biosensors for use in surface-enhanced Raman scattering (SERS). This is due to their good biocompatibility and feasibility [26,27,28,29,30,31]. In this study, as illustrated in Scheme 1, we show that the SERS-based boric acid nanoprobe is an effective tool for intracellular ROS measurements [32]. We used a SERS boronic-acid-based dual-parameter monitoring nanoprobe for taking simultaneous intracellular ROS and PI3K/Akt measurements during ginsenoside Rg3-induced cell apoptosis.

## 2. Materials and Methods

### 2.1. Reagents and Instruments

P-Mercapto benzenboric acid (4-MPBA) and AuNRs were purchased from Beijing Putian Tongchuang Biotechnol (Beijing, China) and Nanoseedz Corp (Hong Kong, China), respectively. The CCK-8 (Cell Counting Kit-8) was obtained from Dojindo (Kumamoto Prefecture, Japan). The phosphate buffer (PBS) and fetal bovine serum (FBS) were obtained from Hyclone (Logan, UT, USA). Dulbecco’s Modified Eagle Medium (DMEM) was obtained from the Roswell Park Memorial Institute (RPMI-1640). Penicillin and streptomycin were obtained from Corning (Corning, NY, USA). The fluorescence probe DCFH-DA and JC-1 were purchased from Sigma (St. Louis, MO, USA). Ginsenosides Rg3 was obtained from Guangzhou Ruishu Biotechnology (Guangzhou, China). DMSO and paraformaldehyde were purchased from Tianjin Zhiyuan Chemical Reagent (Tianjin, China). Deionized water (>18.2 MΩ.cm) was used for all experiments. Hela cells, HepG2 cells, and Jurkat cells were obtained from Sun Yat-Sen University (Guangzhou, China).

A centrifuge (Changsha Xiangzhi Instrument Co., Ltd. Changsha, China) and a medium-high speed HC-2518 centrifuge (Anhui Zhongke Zhongjia Instrument Co., Ltd. Hefei, China) were used. A Renishaw Raman spectrometer, equipped with a 633 nm laser and a 50-objective lens (NA = 0.6) Lycra microscope, was also used, and it was able to detect a spectral range of 600–1800 cm^−1^. All sample data were processed by MATLAB R2017 and Origin 8.6 software. An ultrasonic cleaning machine (JP 020, manufacturer: Shenzhen Jiemeng Cleaning Equipment Ltd.), fluorescence spectrophotometer (F-4600, Hitachi, Tokyo, Japan), fluorescence microscope (Nikon Ti-s, Tokyo, Japan), enzyme-labeled instrument (Bio-Rad, Hercules, CA, USA), and CO_2_ incubator (2406-2, Shel Lab) were used. Moreover, we used a Clean Bench Biosafety Cabinet (Sujing Group Suzhou Antai Air Technology Co., Ltd. Suzhou, China).

### 2.2. Cell Culture and Drug Treatment

Hela cells and HepG2 cells were cultured with DMEM medium, and Jurkat cells were cultured with 1640 medium, containing 1% penicillin-streptomycin solution and 10% FBS in the incubator. The incubator contained 5% CO_2_ at a temperature of 37 °C, and the culture media were changed every 2 to 3 days. Three types of cells in the logarithmic growth phase were inoculated in the dish for 24 h, and a 6-well plate with a density of 1 × 10^6^/mL was used. Then, ginsenosides Rg3 was added and co-cultured for different lengths of time (1 h, 3 h, 6 h, 12 h, and 24 h, respectively).

### 2.3. Synthesis of the SERS Boric Acid Nanoprobe

SERS boric acid nanoprobes were prepared in accordance with the methods in our previous report [32]. First, 200 μL 40 μM MPBA was added to 1 mL AuNRs colloid, vibrated for 5 min, and then left overnight at room temperature. Next, the above mixture was centrifuged twice at 8000 rpm for 8 min to remove the unreacted components. The prepared nanoprobes were then stored at room temperature for later use.

### 2.4. Biocompatibility of the SERS Boric Acid Nanoprobe

The Hela cells, HepG2 cells, and Jurkat cells in the logarithmic growth phase, at concentrations of 1 × 10^5^/mL, were inoculated in the 96-well plates (100 μL cell suspension in each well), respectively. After 10 μL AuNRs (30 μg/mL) were added and the cells were co-cultured for different lengths of time (1 h, 3 h, 6 h, 12 h, 24 h), 10 μL CCK-8 solution was added and cultured in 37 °C, 5% CO_2_ incubator for 2 h. For comparison, each type of cells not containing AuNRs and only containing PBS solution were used as the control group and blank group, respectively.

### 2.5. Optimum Rg3 Concentration and Time

To determine the optimum Rg3 concentration, we carried out a concentration gradient experiment. Hela cells, HepG2 cells, and Jurkat cells in a logarithmic growth phase, at a concentration of 1 × 10^5^/mL were inoculated in separate 96-well plates (100 μL cell suspension in each well). Rg3 with different concentrations and CCK-8 solution (10 μL each well) were added to the cells, which were then incubated for 2 h (37 °C and 5% CO_2_). Finally, the absorbance was measured using an enzyme-labeled instrument (Bio-Rad, USA), so that the survival rate could be calculated. Consequently, we were able to obtain the optimum drug concentration.

Using the optimum concentration, we set up a time gradient experiment for drug treatment. Similar to the above concentration gradient group, the three types of cells in the logarithmic growth phase, at a concentration of 1 × 10^5^/mL, were inoculated in the 96-well plates (100 μL cell suspension in each well). Rg3 at a certain concentration and CCK-8 solution (10 μL each well) were added to the cells, which were then incubated for 2 h (37 °C and 5% CO_2_). Finally, the absorbance was measured using the enzyme-labeled instrument (Bio-Rad, USA), so that the survival rate could be calculated. Consequently, we were able to obtain the optimum drug treatment time.

### 2.6. Raman Spectrum Measurement

Hela cells and HepG2 cells were inoculated in a 6-well plate with an aluminum sheet for 24 h, and then 60 μL colloid of boric acid nanoprobes with concentration of 65 μg/mL was added after the cells adhered to the bottom of the well. Jurkat cells were directly inoculated in the 6-well plate, and 60 μL of boric acid nanoprobe solution with concentration of 65 μg/mL was added to the wells. After endocytosis for 12 h, the cells were treated with Rg3 and left for different lengths of time (1 h, 3 h, 6 h, 12 h, 24 h). Next, the culture medium was discarded, the cells were washed once with PBS, fixed with paraformaldehyde for 20 min, washed once with PBS and then twice with deionized water. After that, the corresponding Raman spectra of the three types of cells were measured 8–10 times for each cell, and 4–5 cells of each sample were used. Consequently, we can obtain the ROS level by observing the Raman peak intensity ratio of boric acid probes.

### 2.7. Intracellular ROS Measurement with a DCFH-DA Kit

To verify the ROS measurement of the SERS boric acid nanoprobe which can obtain Raman signal enhancement with AuNRs, we used a DCFH-DA kit to measure the intracellular ROS for Hela cells, HepG2 cells, and Jurkat cells, respectively. After Rg3 was used to treat the cells over different lengths of time (1 h, 3 h, 6 h, 12 h, 24 h), the cells were collected, washed once with PBS, stained with 10 µM DCFH-DA, and incubated for 30 min (37 °C and 5% CO_2_). Then, the medium was removed, and the cells were again washed twice with PBS. Finally, the DCF fluorescence intensity was measured using a fluorescence spectrophotometer (F-4600, Hitachi, Japan) within 20 min.

### 2.8. Intracellular PI3K/Akt Measurement

To verify the PI3K/Akt measurement taken by the SERS boric acid nanoprobe, we used a PI3K kit and Akt kit to measure the intracellular PI3K and Akt for Hela cells, HepG2 cells, and Jurkat cells, respectively. The three types of cells in the logarithmic growth phase were inoculated in the dish, and a 6-well plate with a density of 1 × 10^6^/mL was used; the cells were subsequently left for 24 h. Then, ginsenosides Rg3 was added, and the cells were co-cultured for different lengths of time (1 h, 3 h, 6 h, 12 h, 24 h). After the drug treatment, the cells were destroyed by repeatedly freezing and thawing them; the intracellular liquid was then extracted as the sample, and the absorbance rate at 450 nm was measured with a microplate reader (Bio-Rad, USA).

### 2.9. Intracellular MMP Measurement

Mitochondrial membrane potential (MMP), a parameter for characterizing cell apoptosis, was measured with an MMP assay kit, with JC-1, in three types of cells. After Rg3 treatment was used to treat the cells over different lengths of time (1 h, 3 h, 6 h, 12 h, 24 h), the cells were collected and washed once with PBS, stained with JC-1, and incubated for 30 min (37 °C and 5% CO_2_). Next, the medium was removed, and the cells were washed twice again with PBS. Finally, the stained cells were examined using a fluorescence spectrophotometer (F-4600, Hitachi, Japan) within 20 min.

## 3. Results and Discussion

### 3.1. Cell Viability Assay

**Figure 1:**
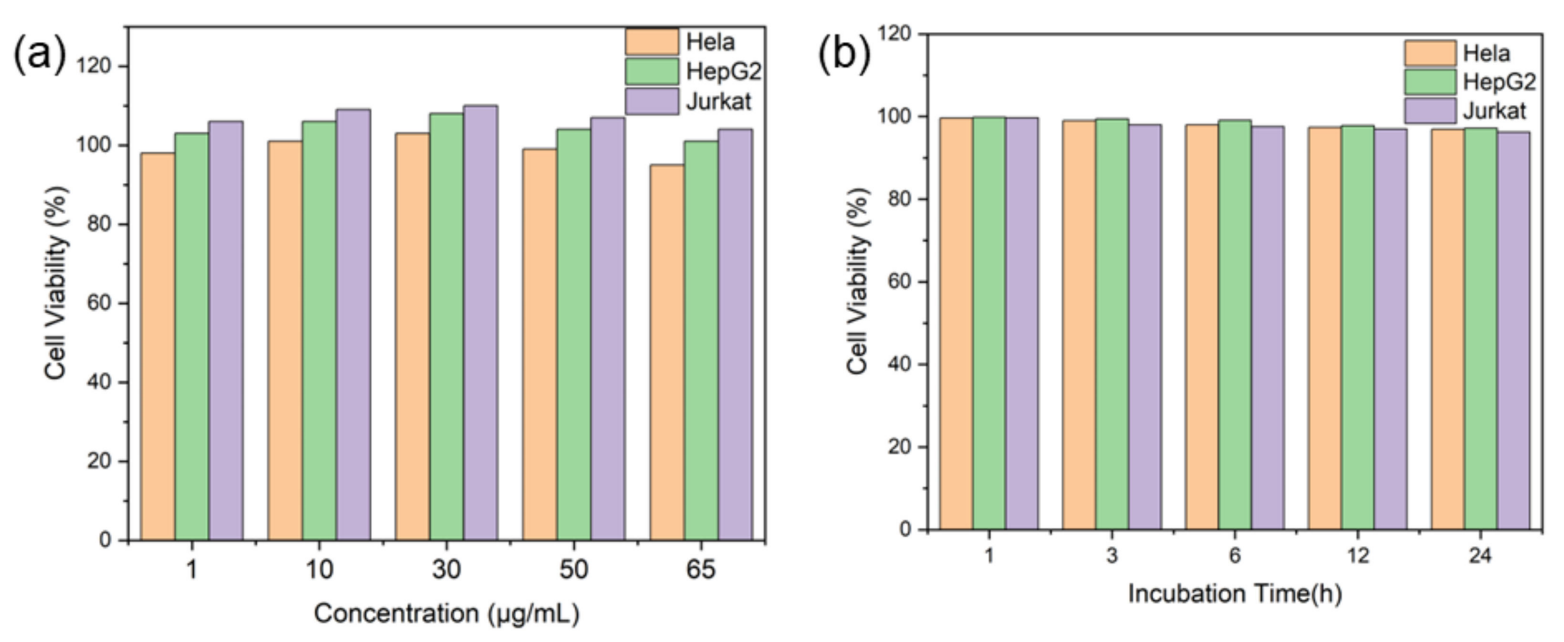
Survival rates of three types of cells co-cultured with SERS boric acid nanoprobes at different (**a**) concentrations (1–65 μg/mL) and (**b**) times.

As with our previous study, Cell Counting Kit-8 (CCK-8) was used to examine the survival rate of Hela cells, HepG2 cells, and Jurkat cells that were co-cultured with a SERS boric acid nanoprobe. As shown in Figure 1, the survival rates of the three types of cells are greater than 90% at different concentrations (1–65 μg/mL) and incubation times, thus indicating that the SERS boric acid nanoprobes have a good biocompatibility and low toxicity.

### 3.2. Optimum Rg3 Concentration

**Figure 2:**
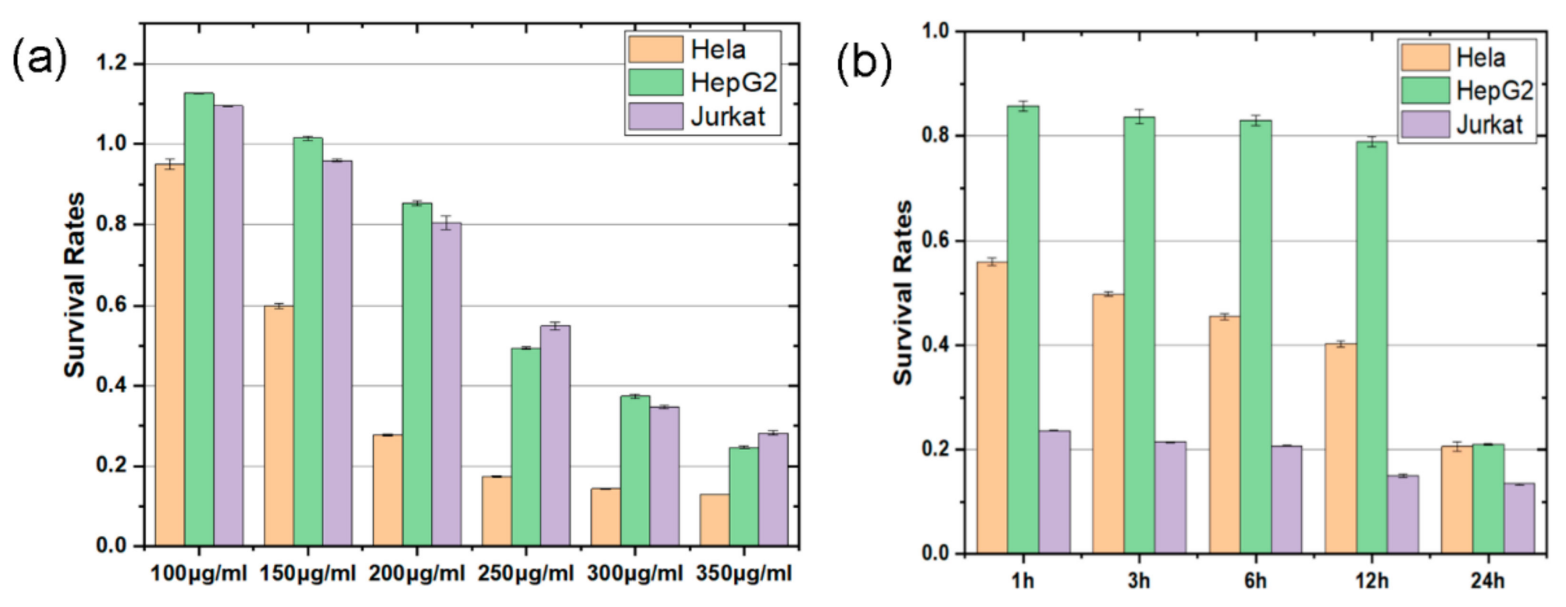
Survival rates of three types of cells treated with Rg3 at different (**a**) concentrations and (**b**) times.

To determine the optimum Rg3 concentration, we set up a concentration gradient experiment to examine cell viability. First, three types of cells were treated with Rg3 at different concentrations of 100, 150, 200, 250, 300, 350 μg/mL. Then, the survival rate was examined after the cells had been treated with Rg3 for 24 h, as shown in Figure 2a. It is evident that the survival rates of the three types of cells diminished as Rg3 concentration increased, particularly with regard to Hela cells. When the Rg3 concentration increased to 200 μg/mL, the survival rate of the Hela cells was almost unchanged; therefore, we chose 200 μg/mL as the experimental Rg3 concentration.

As shown in Figure 2b, the survival rates of all three types of cells decreased as the Rg3 treatment time increased. Moreover, the effect of Rg3 in the HepG2 cells was weak, but it was strong in the Jurkat cells. In addition, the effect that Rg3 has in Hela cells is time-dependent. These results indicate that Hela cells are good candidates for studying the regulatory effect of the ROS/PI3K/Akt pathway during Rg3-induced cell apoptosis.

### 3.3. Intracellular ROS Measurements Using a SERS Nanoprobe

**Figure 3:**
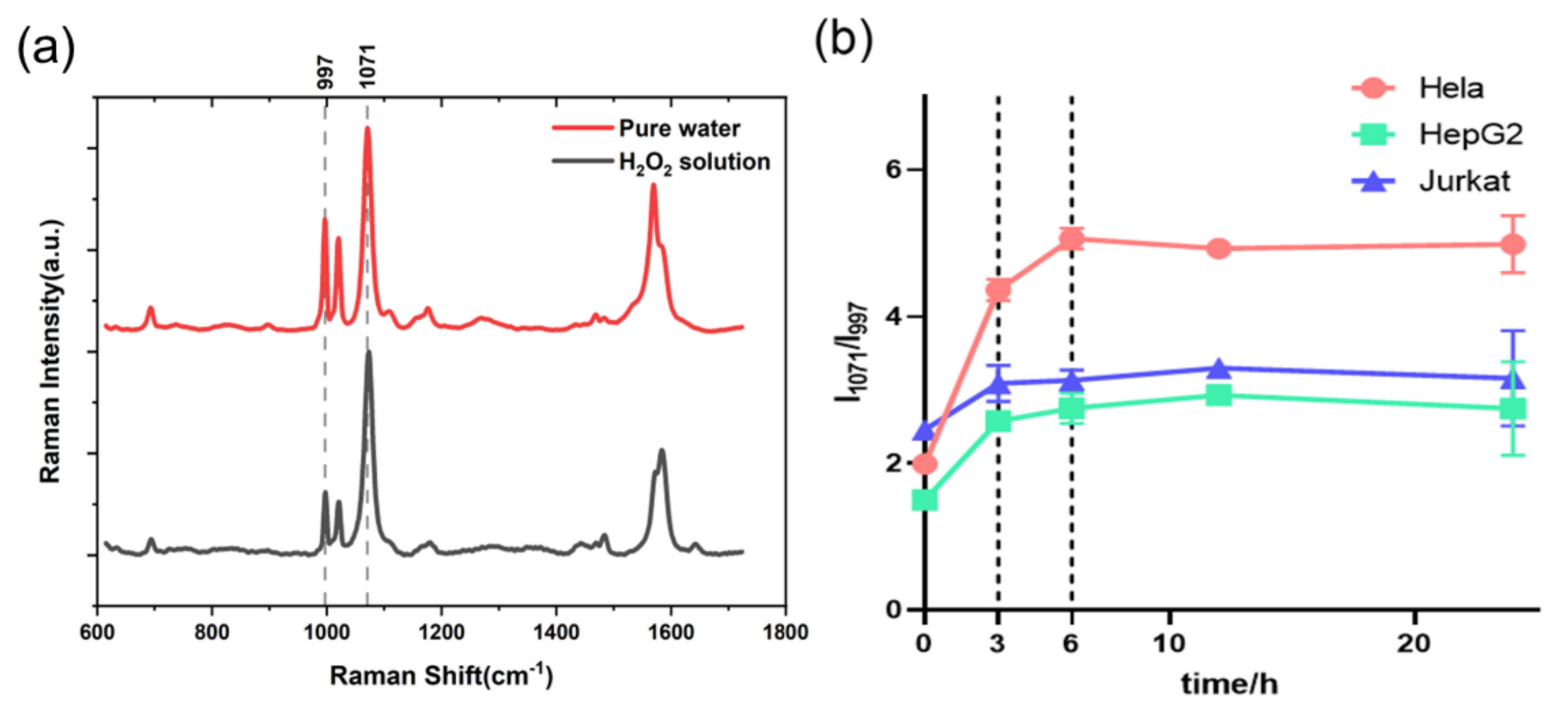
(**a**) Raman spectra of the SERS boric acid nanoprobe measured in H_2_O_2_ solution and pure water solution without H_2_O_2_; (**b**) the change in Raman peak intensity ratio I_1071_/I_997_ of three types of cells versus time.

In accordance with a previous study, the Raman peaks were measured at 1071 cm^−1^ and 997 cm^−1^, respectively. These peaks were attributed to the plane deformation of C-H on the benzene ring and the symmetrical B-O stretching vibration. As the SERS boric acid nanoprobe can be specifically bound to H_2_O_2_, the reaction between ROS nanoprobe and H_2_O_2_ will lead to the intensity reduction or disappearance of ROS nanoprobe Raman peak attributed to B-O symmetric stretching at 997 cm^−1^. Meanwhile, the benzene ring does not participate in the reaction, so the peak intensity of the deformation in the C-H plane attributed to the benzene ring at 1071 cm^−1^ remains unchanged; thus, we can conveniently determine the intracellular ROS level using the Raman peak intensity ratio I_1071_/I_997_ (Figure 3a). Especially since the Raman peak intensity ratio of I1071/I997 is used to identify the reaction occurring, it is not necessary to ensure the Raman spectrum at 1071 cm^−1^ as 0 at the initial stage. Figure 3b shows the change in the Raman peak intensity ratio I_1071_/I_997_ versus time in the three types of cells. Before 6 h had passed, the ROS level in the Hela cells rapidly increased, and the ROS level in the HepG2 cells and Jurkat cells increased until 12 h had passed. Then, the ROS level rapidly increased in the first 3 h, and continued to gradually increase afterwards. Moreover, the intensity ratio I_1071_/I_997_ that exhibited the maximum change appeared in the Hela cells, thus indicating that ROS production during Rg3-induced cell apoptosis was closely related to cell type.

### 3.4. Raman Spectrum Analysis during Rg3-Induced Cell Apoptosis

**Figure 4:**
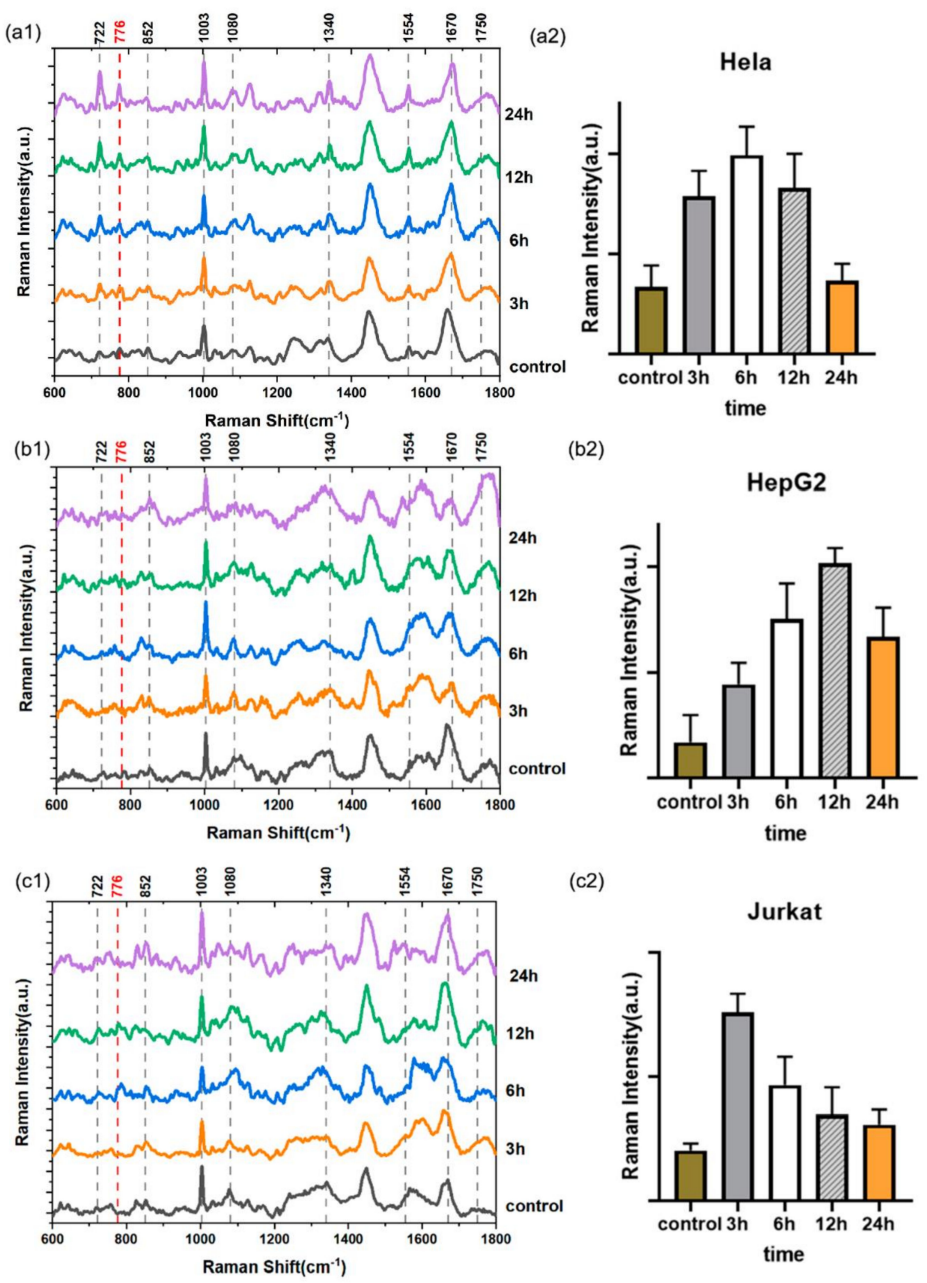
(**a1**–**c1**) Raman spectra of the three Rg3−treated cell types over different lengths of time; (**a2**–**c2**) the normalized intensity of the peak at 776 cm^−1^ versus time.

Figure 4(a1–c1) show the Raman spectra of three types of cells treated with Rg3 at different times. As shown in Table 1, the change in Raman peaks in the three types of cells were mainly caused by the vibrations and deformations of nucleic acids, proteins, and lipids. The peaks at 776, 1670, and 1750 cm^−1^ were attributed to the lipids, the peaks at 722 and 1080 cm^−1^ were attributed to DNA, and the peaks at 852, 1003, 1080, and 1554 cm^−1^ were attributed to proteins. After the cells were treated with ginsenoside Rg3 for 24 h, most of the peaks had significantly decreased in intensity, thus indicating that the cell division cycle entered the G2 phase known as the mitosis preparation period. This meant that the cells had begun to synthesize proteins, nucleic acids, lipids, and other substances before the cells started to divide and proliferate.

In addition to these main Raman peaks—which are attributable to changes in DNA, protein, and carbohydrates (Table 1)—we also focused on the intensity with which the peak at 776 cm^−1^ changed. The intensity of the peak change was attributed to the addition of phosphatidylinositol, and it highlighted the regulatory effect of the PI3K/Akt pathway, as shown in Figure 4(a2–c2). Moreover, the phosphatidylinositol content in the three types of cells first increased and then decreased after Rg3 treatment. As PI3K usually catalyzes the production of phosphatidylinositol-3,4,5-triphosphate (PI(3,4,5)P_3_), after the PI3K was activated, Akt was recruited from the cytoplasm to the plasma membrane as a result of its interaction with PI(3,4,5)P_3_. This led to conformation changes, and it activated the phosphorylation of two residues [33].

**Table 1:** Raman spectral interpretations [34].

Raman Shift/cm^−1^	Assignments	Components
722	Deoxyribonucleic acid	DNA
776	Phosphatidylinositol	Lipids
852	Proline Hydroxyproline Tyrosine Glycogen	Protein
1003	Phenylalanine, C-C skeletal	Protein
1080	Typical phospholipids Phosphodiester groups in nucleic acids Collagen	Protein DNA
1340	Nucleic acid	DNA
1554	Amide II Tryptophan	Protein
1670	Amide I C=C stretching vibrations Cholesterol and its esters	Protein Lipids
1750	C=O of lipids	Lipids

Subsequently, the change in PI(3,4,5)P_3_ content, as measured by the Raman spectrum, can be explained as follows. At the beginning of the Rg3 treatment, PI3K was not inhibited, and PI(3,4,5)P_3_ was produced in order to enable the promotion of the PI3K/Akt pathway. After 6 h of Rg3 treatment in Hela cells, 12 h of treatment in HepG2 cells, and 3 h of treatment in Jurkat cells, the activity of PI3K was inhibited. The PI(3,4,5)P_3_ content was also subsequently reduced, thus inhibiting the PI3K/Akt pathway. Combined with the above analysis in Figure 3b, we can conclude that after Rg3 treatment for 6 h, in Hela cells and HepG2 cells, the PI3K/Akt pathway was promoted as the ROS level increased. For Jurkat cells, this pathway was promoted in the first 3 h, and then it was inhibited as the ROS level increased, thus indicating that the regulatory effect of the ROS level on the PI3K/Akt pathway was closely related to cell types.

### 3.5. PI3K/Akt Measurement

**Figure 5:**
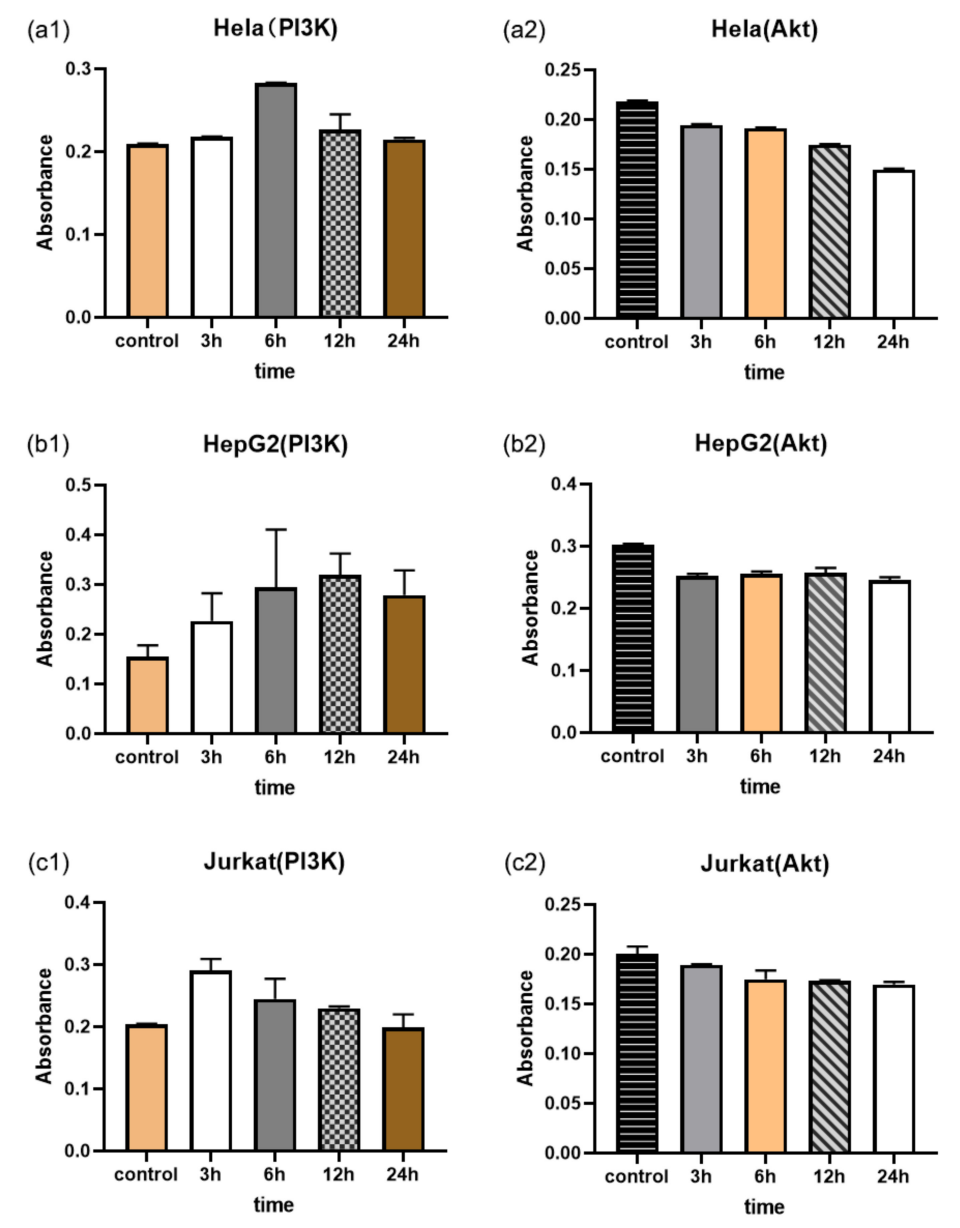
PI3K and Akt contents in the three Rg3-treated cell types, as ascertained by the absorbance measurements obtained using the PI3K and Akt kits; (**a1**–**c1**) PI3K measurements; (**a2**–**c2**) Akt measurements.

For cell survival and phosphorylation, the promotion of Akt is usually considered a key factor in cancer invasion, and Akt activation also leads to tumor metastasis [11]. Ginsenoside Rg3-induced apoptosis was accompanied by the downregulation of PI3K/Akt family proteins [35]. Consequently, we employed PI3K and Akt kits in order to verify the measurement of the Raman boric acid nanoprobes. Figure 5(a1–c1,a2–c2) show the PI3K and Akt contents of the three types of cells after Rg3 treatment, respectively. It is evident that the PI3K content in the in the three types of cells first increased and then decreased. As PI3K catalyzed the production of PI(3,4,5)P_3_, the change in PI3K content also indicated that the PI(3,4,5)P_3_ content first increased, then decreased. When PI3K was activated, Akt was recruited from the cytoplasm to the plasma membrane in response to the interaction with PI(3,4,5)P_3_. A downward trend in Akt content indicated that this was a response to PI(3,4,5)P_3_ inhibition. Collectively, these results reveal that the PI3K/Akt pathway was first promoted and then inhibited, which was consistent with our dual-parameter monitoring probe measurement results.

### 3.6. Intracellular ROS Measurement Using a DCFH-DA Probe

**Figure 6:**
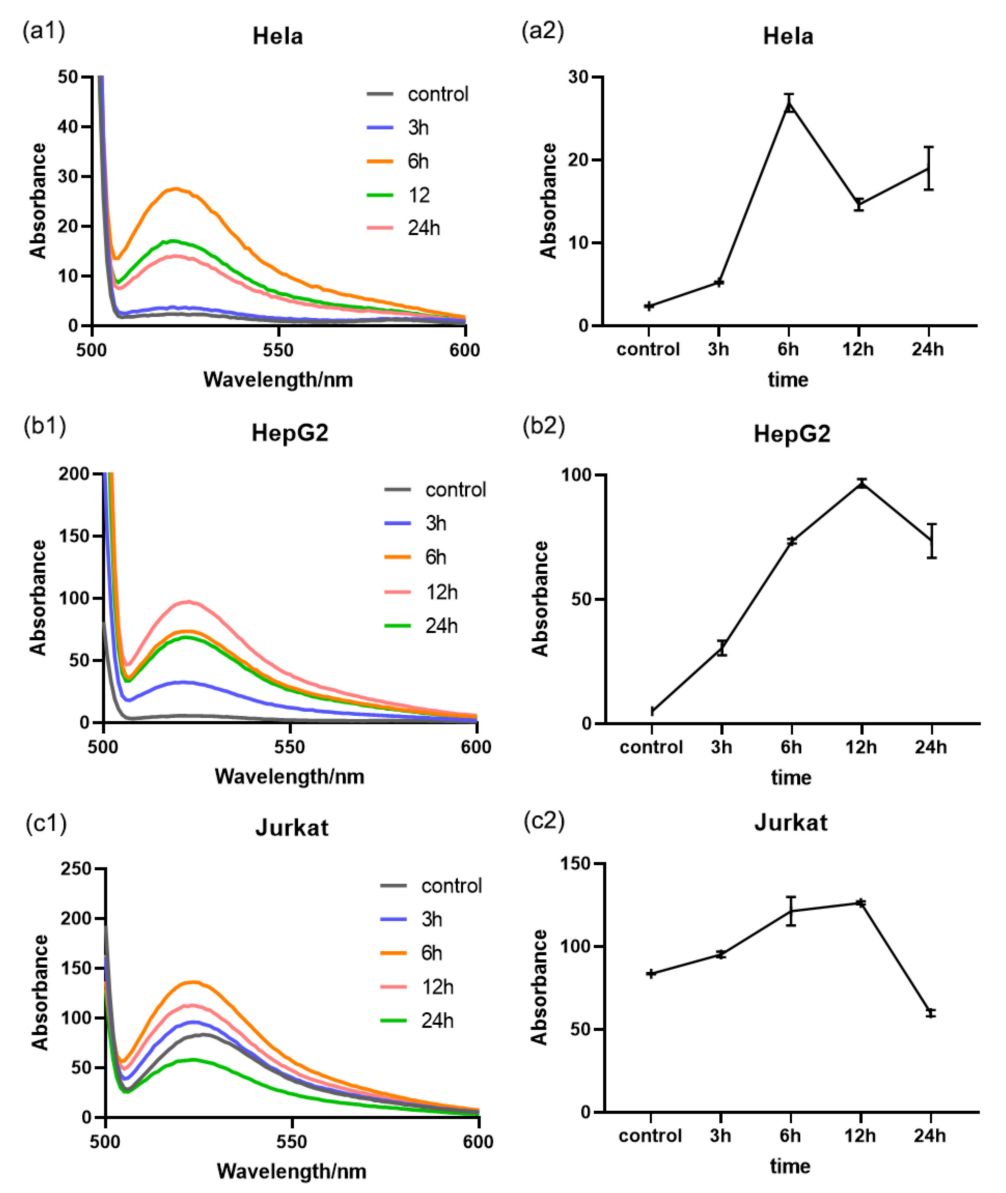
(**a1**–**c1**) intracellular ROS levels of the three Rg3-treated cell types, as determined using the absorbance measurements taken with the DCFH-DA probe; (**a2**–**c2**) intracellular ROS level changes versus treatment time.

To verify the effectiveness of the SERS boric acid nanoprobe, a ROS kit known as DCFH-DA fluorescent probe was used to measure the ROS level. First, we set up a time gradient experiment to examine the intracellular ROS level after Rg3 treatment had taken place over 3, 6, 12, and 24 h, as shown in Figure 6. Different from the above SERS nanoprobe measurement result (Figure 3), the ROS level measured by the DCFH-DA probe decreased after reaching the maximum value. This is because the reaction between the nanoprobes and ROS was irreversible; indeed, when the ROS decreased, the intensity of the Raman peak at 997 cm^−1^, which may be attributed to the B-O bond, did not increase and the intensity ratio of I_1071_/I_997_ did not change. Consequently, the ROS level in Hela cells reached a maximum after Rg3 treatment for 6 h in HepG2 and 12 h in Jurkat cells; these results were consistent with the trend of intensity ratio I_1071_/I_997_ of SERS. Accordingly, the ROS level in Hela cells experienced the greatest amount of change, relative to the other two types of cells. Clearly, this result was consistent with our SERS boric acid nanoprobe measurement.

### 3.7. Mitochondrial Membrane Potential Measurement

**Figure 7:**
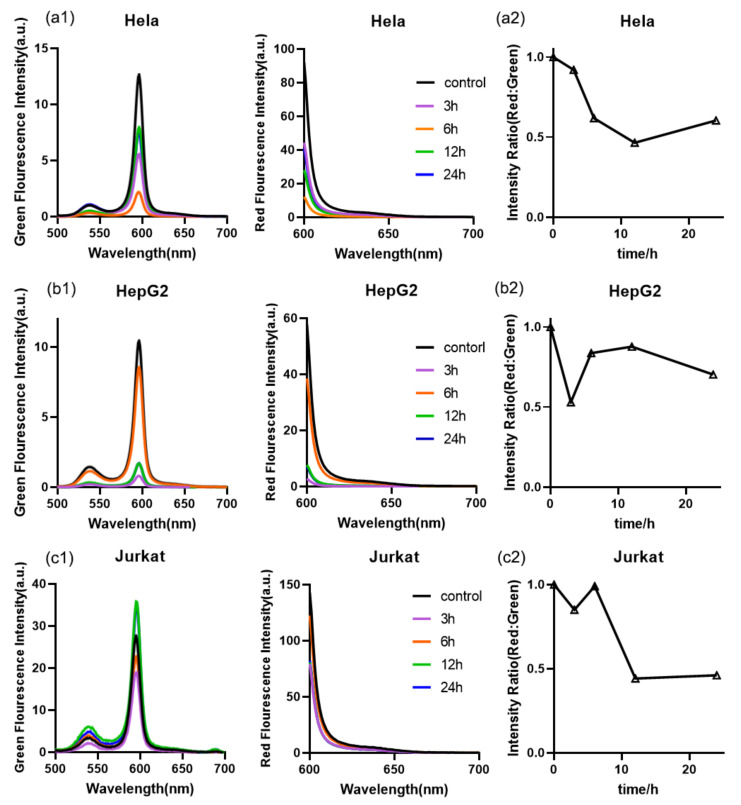
(**a1**–**c1**,**a2**–**c2**) MMP of the three Rg3-treated cell types, as determined using green and red fluorescence spectral measurements taken with the MMP assay kit and JC-1.

Mitochondrial membrane potential (MMP), a typical parameter for characterizing cell apoptosis, was used as the reference for analyzing intracellular ROS levels during Rg3-induced cell apoptosis. Mitochondrial-mediated apoptosis was implemented by increasing the permeability of the mitochondrial membrane [17,36]. Rg3 exerted a pro-apoptotic effect to cause the reduction in MMP; once the MPP was lost, the cell entered an irreversible apoptotic process. The unlinked respiratory chain of mitochondria generated a large amount of ROS, which oxidized phospholipids on the inner mitochondrial membrane and reduced energy production, thus causing a chain reaction that further increased ROS level. From Figure 7(a1,a2), it is evident that in the first 6 h of Rg3 treatment, the MMP in the Hela cells significantly decreased, whereas the intracellular ROS level greatly increased. Similarly, the MMP in the HepG2 cells and Jurkat cells decreased during the first 3 h, whereas the ROS levels significantly increased. Combined with the change in intracellular ROS level, we found that the MMP was significantly reduced during cell apoptosis, whereas the ROS level rapidly increased.

## 4. Conclusions

In this study, we examined a SERS-based boric acid nanoprobe to simultaneously monitor the intracellular ROS level and PI(3,4,5)P_3_ content in order to demonstrate the PI3K/Akt pathway’s regulatory effect during ginsenoside Rg3-induced cell apoptosis. The Raman peak intensity ratio I_1071_/I_997_ was used to calculate the intracellular ROS level, and the PI(3,4,5)P_3_ content was examined using the Raman peak at 776 cm^−1^. Our results show that in the three types of cells (Hela, HepG2, and Jurkat), the ROS levels increased within the timeframe wherein Rg3 treatment took place, whereas the PI(3,4,5)P_3_ content first increased, then decreased after Rg3 treatment, thus indicating that the PI3K/Akt pathway was first promoted and then inhibited. The peak intensity changes in PI(3,4,5)P_3_ show that the regulatory effect of the PI3K/Akt pathway is closely related to the ROS level and cell type. Along with the increase in Rg3 treatment time, the PI3K/Akt content level increased as the ROS increased, and when the ROS level increased significantly, the MMP significantly reduced. Different from the previous reports concerning ROS accumulation, and how it leads to PI3K/Akt pathway inhibition, our experimental result demonstrates that a low ROS level also helps to promote the PI3K/Akt pathway. Importantly, in addition to avoiding evaluation contingency, which is measured by different techniques, this SERS-based dual-parameter monitoring nanoprobe provides an effective solution for simultaneous ROS level and PI3K content measurements during cell apoptosis, thus enabling the acquisition of dynamic regulatory information concerning the intracellular ROS level on the PI3K/Akt pathway; this is imperative for studying ROS/PI3K/Akt-pathway-related cell apoptosis and activation mechanisms.

## Data Availability

Not applicable.
